# The entorhinal cortex is involved in conditioned odor and context aversions

**DOI:** 10.3389/fnins.2015.00342

**Published:** 2015-10-02

**Authors:** Barbara Ferry, Karine Herbeaux, Hervé Javelot, Monique Majchrzak

**Affiliations:** ^1^Centre of Research in Neuroscience, CNRS UMR 5292 - INSERM U1028 - UCBL1Lyon, France; ^2^Laboratoire de Neurosciences Cognitives et Adaptatives, UMR 7364, Faculté de Psychologie, CNRS, Université de Strasbourg, Neuropôle de Strasbourg, GDR 2905 du CNRSStrasbourg, France; ^3^Etablissement Public de Santé Alsace Nord Brumath – Service Pharmacie - CHU de Strasbourg, Hôpital Civil, Clinique de Psychiatrie – Service de Psychiatrie IIBrumath, France

**Keywords:** entorhinal cortex, odor aversion, context aversion, food conditioning, odors

## Abstract

In a natural environment, avoidance of a particular food source is mostly determined by a previous intake experience during which sensory stimuli such as food odor, become aversive through a simple associative conditioned learning. Conditioned odor aversion learning (COA) is a food conditioning paradigm that results from the association between a tasteless scented solution (conditioned stimulus, CS) and a gastric malaise (unconditioned stimulus, US) that followed its ingestion. In the present experimental conditions, acquisition of COA also led to acquisition of aversion toward the context in which the CS was presented (conditioned context aversion, CCA). Previous data have shown that the entorhinal cortex (EC) is involved in the memory processes underlying COA acquisition and context fear conditioning, but whether EC lesion modulates CCA acquisition has never be investigated. To this aim, male Long-Evans rats with bilateral EC lesion received CS-US pairings in a particular context with different interstimulus intervals (ISI). The results showed that the establishment of COA with long ISI obtained in EC-lesioned rats is associated with altered CCA learning. Since ISI has been suggested to be the determining factor in the odor- and context-US association, our results show that the EC is involved in the processes that control both associations relative to ISI duration.

## Introduction

Finding adequate food sources', including water, is one of the most fundamental aspects of animal life. Guided by various kinds of stimuli present in the environment, animals move through space toward a resource goal, avoiding unsafe environments and the risk of themselves becoming food. This behavior involves the animal finding and recognizing particular cues, whether contingent, in a specific environment, or coming directly from the food source, that indicate the nearness and direction of the goal. Although innate, a lot of encounters with cue stimuli result in learned approaches or avoidances. In this situation, the capacity to anticipate the future and differentiate between safe or unsafe food items depends, at least in part, on previous experience in which the sensory stimuli characterizing a particular food (odor and taste) become associated with the positive (energy input) or negative (gastric malaise, poisoning) consequences of ingestion through Pavlovian associative learning (e.g., Mehiel and Bolles, 1984; Capaldi et al., 1987; Fedorchak and Bolles, 1987; Harris et al., 2000). These kinds of association have been largely studied (Slotnick and Katz, 1974; Nigrosh et al., 1975; Slotnick, 1984) and the use of conditioned food aversion paradigms in research has provided fundamental insights into the brain mechanisms and structures involved in food-reward/food-poisoning associations (see Miranda, 2012 for a review). One such paradigm, conditioned odor aversion (COA), consists in avoidance of an odorized-tasteless solution (conditioned stimulus, CS) the ingestion of which precedes toxicosis (unconditioned stimulus, US). COA is a robust and long-lasting learned association that can be obtained with a single CS-US pairing (Lorden et al., 1970; Hankins et al., 1973; Taukulis, 1974), and with CS-US delays (interstimulus interval, ISI) ranging from minutes to hours depending on whether the CS is mixed (proximal presentation, inducing orthonasal, and retronasal stimulations: Slotnick et al., 1997; Chapuis et al., 2007) or presented close to the solution (distal presentation, inducing orthonasal stimulation: Garcia et al., 1966; Andrews and Braveman, 1975; Palmerino et al., 1980; Bouton et al., 1986; Ferry et al., 1996, 2006).

Many studies of the mechanisms and systems involved in the memory processes underlying COA learning have focused exclusively on conditioned responses toward the CS. In particular, some showed that the entorhinal cortex (EC) plays an important role in task acquisition. The EC is a parahippocampal structure that receives important olfactory projections (Krettek and Price, 1977; Haberly and Price, 1978; Burwell and Amaral, 1998) and is reciprocally connected to the hippocampus and amygdala (e.g., Amaral and Witter, 1995; Ferry et al., 1997; Pitkänen et al., 2000). In addition, electrophysiological data suggest that the processing of olfactory information in both these regions is modulated by the EC (e.g., Biella and de Curtis, 2000; Gnatkovsky et al., 2004; Mouly and Di Scala, 2006). Interestingly, we found that rats with EC lesions were able to learn to avoid an odor paired with toxicosis, using both distal and proximal CS presentation, even when the ISI was too long (120 min) for such learning to be observed in control animals (Ferry et al., 1996, 1999, 2006, 2007). Further experiments suggested that this facilitation of COA with long ISI may be the consequence of lesion-induced suppression of an inhibitory influence of the EC on brain areas involved in olfactory information processing, such as the basolateral amygdala (BLA; Ferry and Di Scala, 1997; Ferry et al., 1999; Mouly and Di Scala, 2006). Also and non-exclusively, persistence of the odor memory trace supporting COA with long ISI in EC-lesioned rats may involve altered processing of other cues present during conditioning. In concordance with this, several studies have shown that the EC is involved in the processing of information related to the context in which the CS-US association was established, mainly during fear conditioning (Maren and Fanselow, 1997; Ji and Maren, 2008; Majchrzak et al., 2006; but: Phillips and LeDoux, 1995; Good and Honey, 1997; Bannerman et al., 2001; Hales et al., 2014). However, although contextual cues contingent to a specific environment are indicators that also contribute to the behavioral response toward a food source, the relationship between contextual and odor cues during conditioned food aversion learning has been little studied. Context aversion has be-12mmen shown to occur concurrently to COA (Hatfield et al., 1992), but whether EC lesion modulates context aversion has never be investigated during COA.

The aim of the present experiment was to assess whether the establishment of COA with long ISI in EC-lesioned rats was associated with altered contextual information processing. To this end, acquisition of COA and conditioned context aversion (CCA) were tested in EC-lesioned animals using two different procedures. The first (COA), consisted in presenting an olfactory cue (odorized water, CS) in a particular context that was followed by LiCl-induced gastric malaise (US), with short, or long ISI. This forward arrangement between CS and context has previously been shown to result in odor and context aversion (Hatfield et al., 1992). The second (CCA), consisted in administering the US after the animals had been placed in a particular context, with the same short and long ISIs. In this procedure, the CS was presented after the US at the end of the session. This backward arrangement between the two stimuli resulted in acquisition of context aversion but not in COA.

## Materials and methods

### Subjects

One hundred and two male Long-Evans rats (supplied from Janvier Labs, Le Genest-St-Isles France; weighing 250–275 g) were used. They were housed two per cage in transparent Makrolon cages (42 × 26 × 15 cm) under controlled temperature (22°C ± 2) and standard 12 h light/dark cycle (lights from 7:00 a.m. to 7:00 p.m.) in a colony room. The animals were provided with *ad libitum* access to food and water. After arrival, the animals were allowed to acclimate to the laboratory conditions for a period of 1 week before surgery.

All procedures involving animals and their care conformed to the institutional guidelines, which comply with international laws and policies (directive 2010/63/European Community) and have been approved by the ethics committee of the Université Claude Bernard Lyon 1 (CE2A-55). Permission references were 69–387517 for BF and 67–289 for MM. All other co-authors were under the responsibility of the former.

### Surgery

All surgical procedures were conducted under optimal aseptic, analgesic, and ethical animal care conditions (see Ferry et al., 2014) by those authorized to do so. Rats were anesthetized by i.p. injection of a mix of ketamine (100 mg/kg)/xylasine (10 mg/kg). Following a prophylactic antibiotic treatment (penicillin 0.12 M.U./0.3 ml, i.m.) the rats were given bilateral lesions of the EC by aspiration (*n* = 56) as previously described (Ferry et al., 1999). Sham-lesioned animals were operated similarly but no aspiration was carried out (*n* = 46). Four animals died after surgery (three EC-lesioned and one sham-lesioned). All subjects recovered for 7–11 days after surgery with *ad libitum* access to food and water, and were singly housed until the end of the experiment.

### Test chambers

#### Habituation chambers

Eight chambers (25 × 30 × 35 cm) located in a room adjacent to the vivarium were used for habituation to water consumption. They were made of clear perspex and had a wire mesh floor. The spout of a 25-ml glass tube (Richter tube, Strasbourg, France) could be introduced into the cage through a circular hole on the anterior wall of the chamber, located 2 cm above the floor. Intake was measured by reading to the nearest 0.5 ml, the level of liquid of the tube before and after each session.

#### Conditioning and testing chambers

Four place preference boxes located in a room adjacent to the vivarium were used. Each box was constituted by two large compartments of similar size (45 × 45 × 30 cm, compartments A and B) with distinctive visual and tactile features and a third smaller ship-wooden gray painted transit compartment (36 × 18 × 30 cm) that allowed animals to move between compartments A and B when the sliding door of their back wall was open. Compartment A had three wooden black walls and a floor made of tight and flexible wire mesh. Compartment B had three wooden walls with vertical black and white stripes and a floor made of large and rigid wire mesh. The front wall of both compartments was constituted by clear perspex. The spout of the Richter tube could be introduced into each compartment through a circular hole on the back wall of the chamber, located 2 cm above the floor.

### Behavioral procedure

All experimental sessions were carried out during the light portion of the cycle between 11:00 a.m. and 1:00 p.m. After post-surgical recovery, animals were handled (3 min/day) for 3 days and weighed daily to verify their adaptation to the deprivation schedule.

On the first day (Day 1), each animal was placed in the transit compartment of the place preference box with sliding doors open, and allowed to move freely in all compartments during a 15 min session. The time spent in each compartment was recorded. The compartment in which the animals spent more time was chosen as the conditioning context, and the other one was chosen as the neutral context.

Water bottles were removed from the home cage in the evening of Day 1 and a 23 h 45 min water deprivation schedule was initiated. During the water drinking habituation sessions (Day 2 to Day 6), rats had access to water once a day according to the following procedure: On Day 2 and 3 the animals had access to water in their home cage for 30 min on Day 2 and for 15 min on Day 3. From Day 4 to Day 6, animals had access to water for 15 min in the habituation chamber.

On Day 7, each animal was placed in the neutral context equipped with a Richter tube and had access to water for 15 min. Then, the Richter tube was removed and the animal received an i.p. injection of 0.9% NaCl (10 ml/kg). Animals spent an additional period of 60 min in the neutral context.

**Figure 1:**
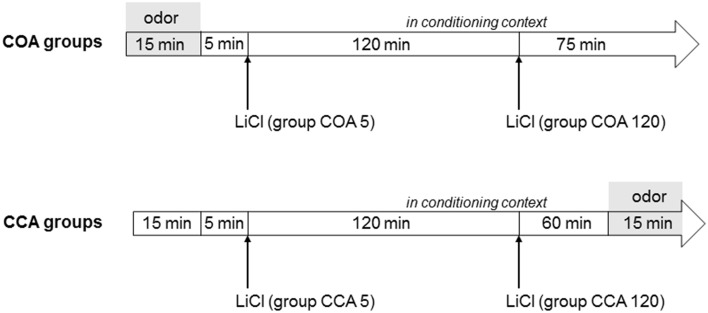
**Time schedule of the conditioning session for COA and CCA groups**. In COA groups, the animals were subjected to presentation of the odor (symbolized by the gray area) in the conditioning context followed by the US with a short (5 min) or a long (120 min) interstimulus interval (COA 5 and COA 120 groups, respectively). The animals spent the rest of the session in the conditioning context. In CCA groups, the animals were placed in the conditioning context for 15 min and then received the US with a short (5 min) or long (120 min) interstimulus interval (CCA 5 and CCA 120 groups, respectively). The animals spent the rest of the session in the conditioning context in which the odor was presented during the last 15 min of the session (symbolized by the gray area). The total duration of the session was 215 min.

Conditioning session took place on Day 8 (Figure 1). Animals were divided in two experimental groups according to the conditioning procedure (adapted from Desmedt et al., 2003). Animals of COA groups were placed in the conditioning context where they had access for 15 min to odorized water in the Richter tube (CS; 0.01% isoamyl acetate solution). At the end of the 15 min, the Richter tube was removed and animals received an i.p. injection of a lithium chloride (LiCl) inducing gastric malaise (US; 0.15 M; 10 ml/kg) either 5 min (short ISI; COA 5 group) or 120 min (long ISI; COA 120 group) after the removal of the Richter tube. Animals spent the rest of the session in the conditioning context (total duration = 215 min).

Animals of the CCA groups were placed in the conditioning context and received an i.p. injection of LiCl at a timing similar to groups COA (either 20 or 140 min after the beginning of the session, referred successively as CCA 5 and CCA 120 groups for convenience). They spent the rest of the 215 min session in the same compartment and had access to the Richter tube filled with 0.01% isoamyl acetate only during the last 15 min of the session. Intake of odorized water was measured by reading the level of liquid on the tube before and after the session in both COA and CCA groups.

EC-lesioned (EC) and sham-lesioned (Sham) rats were randomly assigned to one of these four conditioning procedures. Experimental groups were constituted as follows: COA 5 (EC: *n* = 13; Sham: *n* = 11), COA 120 (EC: *n* = 14; Sham: *n* = 10), CCA 5 (EC: *n* = 13; Sham: *n* = 13), and CCA 120 min (EC: *n* = 13; Sham: *n* = 11).

Conditioned aversions were assessed on Day 9 in the place preference box. To do this, animals were confined in the neutral context with access to the Richter tube filled with 0.01% isoamyl acetate for 15 min. At the end of the session, they returned to their home cages and the amount of liquid consumed during testing was measured. One hour after, animals were placed in the transit compartment with sliding doors open giving access to the two compartments A and B. Time spent in each compartment was measured during 30 min.

### Histology

Ten days after completion of behavioral testing, each rat was given an overdose of sodium pentobarbital (100 mg/kg) and was transcardially perfused with 60 ml of saline (4°C) followed by phosphate-buffered 4% paraformaldehyde (pH 7.4; 4°C). The brain was then extracted, post-fixed for 4 h in the same fixative (4°C) and transferred into a 0.1 M phosphate-buffered 20% sucrose solution for about 36–40 h (4°C). All brains were frozen using isopentane (−40°C). Coronal sections, 30 μm, were cut on a freezing microtome (−23°C), and collected onto gelatine-coated slides. These sections were dried at room temperature and stained with cresyl violet. A microscopic inspection was then performed to determine the location and the extent of the lesions.

### Data analysis

Odor aversion was assessed by comparing the volume of odorized solution intake during the conditioning and testing sessions. Context aversion was assessed by comparing the proportion of time spent in the conditioning context before (pre-conditioning session) and after (testing session) conditioning. For each ISI, the data corresponding to the volumes (for COA) and the ratios (time spent in conditioning context/time of the session) were analyzed with a Three-Way repeated measures ANOVA with Lesion (EC vs. sham), Type of procedure (COA vs. CCA) as between subject factors and Session (conditioning vs. testing for COA assessment, pre-conditioning vs. testing for CCA assessment) as within subject factor. *Post-hoc* Newman-Keuls multiple range test (NK) was used to determine the source of detected significances in the ANOVAs. A probability level of < 0.05 was accepted as statistically significant throughout.

## Results

### Histology

Seventeen EC-lesioned animals (with unilateral damage in the EC or extensive lesions of surrounding structures such as the perirhinal cortex, subiculum, or dentate gyrus) and four sham-lesioned animals with damage to the lateral part of the EC (resulting from the insertion of the curved needle) were excluded from the analysis. Final groups were constituted as follows: COA 5 (EC: *n* = 9; Sham: *n* = 9), COA 120 (EC: *n* = 10; Sham: *n* = 9), CCA 5 (EC: *n* = 9; Sham: *n* = 13), and CCA 120 min (EC: *n* = 8; Sham: *n* = 10).

**Figure 2:**
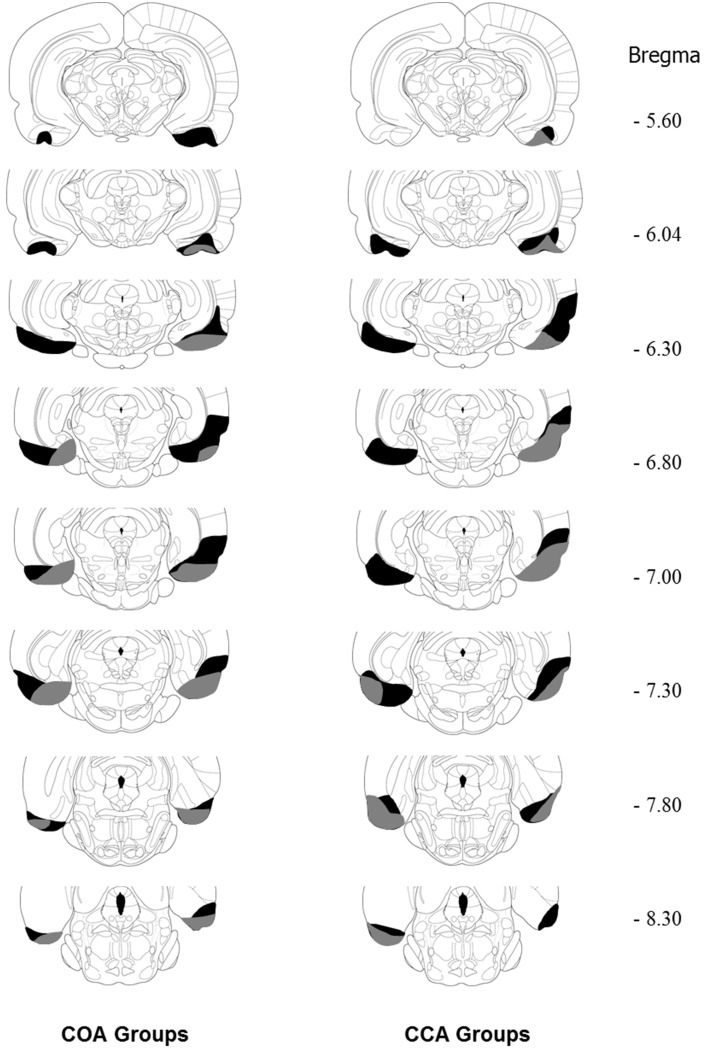
**Diagram of coronal sections (between −5.60 and −8.30 mm from bregma; according to the Paxinos and Watson stereotaxic atlas, 1998) showing the extent of the smallest (gray area) and the largest (black area) EC lesion in the COA and CCA groups**. From *The Rat Brain in Stereotaxic Coordinates*, by Paxinos and Watson (1998). Copyright 2006 by Elsevier. Adapted with permission.

Lesions were drawn from transversal sections stained with cresyl violet. Typical (i.e., smallest and largest) lesion extents observed in COA and CCA groups are illustrated in Figure 2. Histological analysis revealed that all the lesions included the lateral EC at the levels −5.60 to −8.30 from Bregma and a part of the medial EC at Bregma −7.80. The largest lesions were estimated to damage a part of the perirhinal cortex at the levels −6.30 to −7.80 mm.

### Behavior

#### Short ISI-induced conditioned odor and context aversions

**Figure 3:**
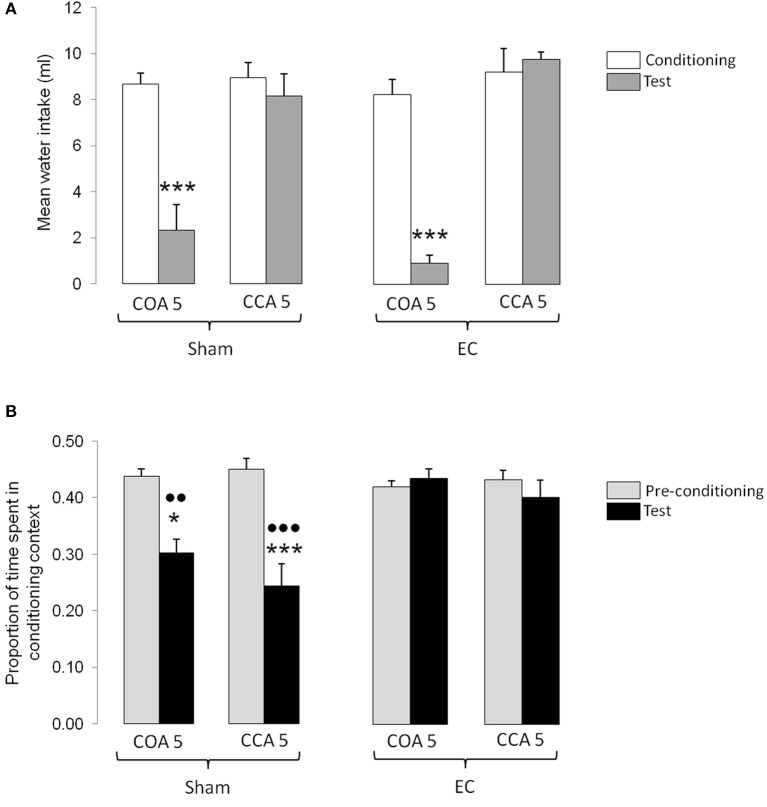
**Effect of a short ISI on COA and CCA in Sham- and EC-lesioned groups**. **(A)** Represents the mean odorized water intakes (± S.E.M.) measured during the conditioning (white bars) and testing (gray bars) sessions in each experimental group. ^***^p < 0.001 as compared with the amount of odorized water intake during conditioning in the same group and with the amount of odorized water intake during testing in the corresponding CCA group. **(B)** represents the mean proportion of time (± S.E.M.) spent in the conditioning context for each experimental group. Bars represent the proportion of time spent in the conditioning context (mean time spent in the conditioning context/time of the session) calculated in the pre-conditioning (gray bars) and testing (black) sessions. ^*^, ^***^p < 0.05 and 0.001 as compared with the proportion of time spent in the conditioning context during pre-conditioning in the same group;
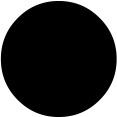

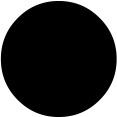
, 
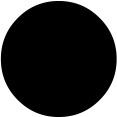

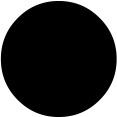

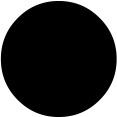
*p* < 0.01 and 0.001 as compared with the proportion of time spent in the conditioning context during testing in the corresponding EC-lesioned-group. EC, lesion of the entorhinal cortex; Sham, sham-lesion of the entorhinal cortex; COA, conditioned odor aversion; CCA, conditioned context aversion.

The results obtained in the experimental groups conditioned with the short ISI are shown in Figure 3. Figure 3A represents the mean odorized water intakes (± S.E.M.) measured during the conditioning and testing sessions whereas Figure 3B represents the mean proportion of time (± S.E.M.) spent in the conditioning context during the pre-conditioning and testing sessions. As shown in Figure 3A, the amount of odorized water intake during conditioning was similar between groups indicating that neither the lesion nor the early intoxication in CCA 5 groups (i.e., 180 min before) had an impact on intake. During testing the amount of odorized water intake was lower in animals exposed to the CS before intoxication, whatever the type of lesion. This indicated that short ISI lead to a COA when the odor is encountered before but not after intoxication. Statistical analyses confirmed these observations and revealed no significant effect of Lesion [*F*_(1, 36)_ = 0.14, p n.s.], a significant effect of Type of procedure [*F*_(1, 36)_ = 55.37, *p* < 0.0001], a significant effect of Session [*F*_(1, 36)_ = 39.39, *p* < 0.0001], and a significant interaction between Session and Type of procedure [*F*_(1, 36)_ = 36.54, *p* < 0.0001]. *Post-hoc* comparisons confirmed that, in both sham- and EC-lesioned COA 5 groups, the odorized water intake during testing was significantly lower than during conditioning (*p* < 0.001 in each case), and also lower than the odorized water intake measured in CCA 5 groups during testing (*p* < 0.001 in each case).

As shown in Figure 3B, the proportion of time spent in the conditioning context during pre-conditioning was similar between groups. In sham-lesioned animals, the proportion of time spent in the conditioning context during testing was lower than before conditioning whatever the type of procedure. This decrease indicated that short ISI lead to a CCA and that animals had associated the context with the US, whether exposed or not to the odorized water. In contrast, the time spent in the conditioning context did not differ between pre-conditioning and testing sessions in EC-lesioned groups, indicating that the lesion affected CCA. The ANOVA confirmed these observations and showed significant effects of Lesion [*F*_(1, 36)_ = 15.13, *p* < 0.001] and Session [*F*_(1, 36)_ = 20.00, *p* < 0.0001] but no effect of Type of procedure [*F*_(1, 36)_ = 1.15, p n.s.], and a significant Session X Lesion interaction [*F*_(1, 36)_ = 16.52, *p* < 0.0001]. *Post-hoc* comparisons confirmed that the proportion of time spent in the conditioning context during testing was lower than before conditioning in both sham-lesioned groups (*p* < 0.05 for COA group and *p* < 0.001 for CCA 5 group), but not in EC-lesioned groups. The proportion of time spent in the conditioning context during testing in sham-lesioned groups was also lower than the proportion of time spent in the conditioning context in EC-lesioned groups (*p* < 0.001 for sham COA 5 vs. EC COA 5 and *p* < 0.001 for sham CCA 5 vs. EC CCA 5).

#### Long ISI-induced conditioned odor and context aversions

**Figure 4:**
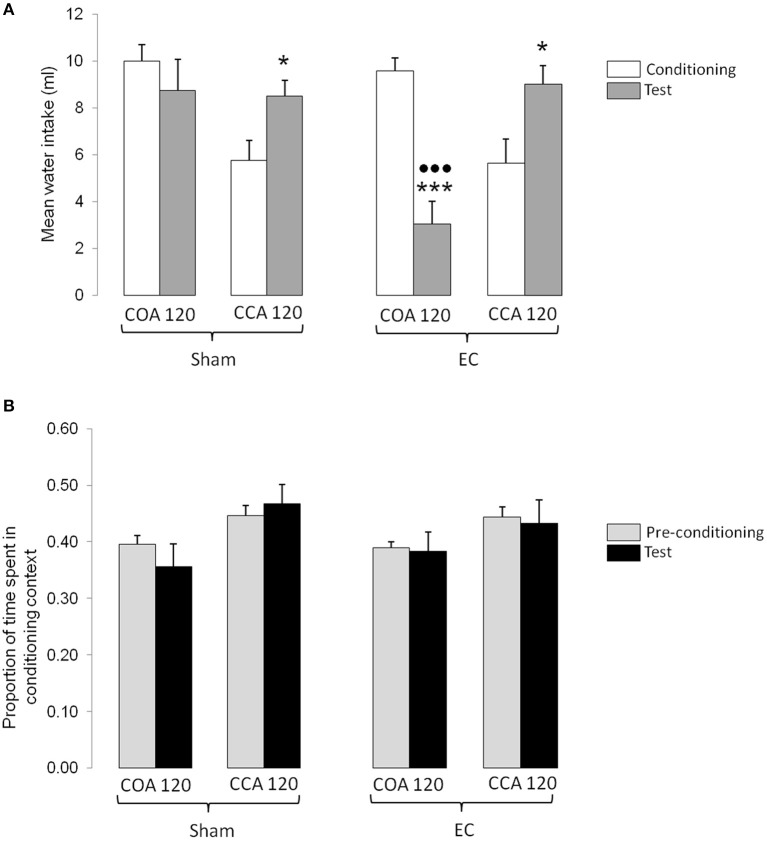
**Effect of a long ISI on COA and CCA in Sham- and EC-lesioned groups**. **(A)** Represents the mean odorized water intakes (± S.E.M.) measured during the conditioning (white bars) and testing (gray bars) sessions in each experimental group. ^*^, ^***^p < 0.05 and 0.001 as compared with the amount of odorized water intake during conditioning; 
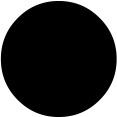

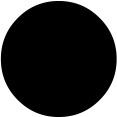

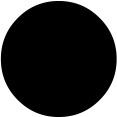
 *p* < 0.001 as compared with the amount of odorized water intake during testing in all the other groups. **(B)** represents the mean proportion of time (± S.E.M.) spent in the conditioning context for each experimental group. Bars represent the proportion of time spent in the conditioning context (mean time spent in the conditioning context/time of the session) calculated in the pre-conditioning (gray bars) and testing (black) sessions. EC, lesion of the entorhinal cortex; Control, sham-lesion of the entorhinal cortex; COA, conditioned odor aversion; CCA, conditioned context aversion.

The results obtained in the experimental groups conditioned with the long ISI are shown in Figure 4. Figure 4A represents the mean water intakes (± S.E.M.) measured during conditioning and testing sessions whereas Figure 4B represents the mean proportion of time (± S.E.M.) spent in the conditioning context during the pre-conditioning and testing sessions. As shown in Figure 4A, the amount of odorized water intake during conditioning was similar in COA 120 groups indicating that the lesion did not affect the level of odorized water intake when the US followed its presentation. However, the amount of odorized water intake during conditioning was lower in CCA 120 groups suggesting an effect of the US when it was administered 60 min before the odorized water presentation. The higher amount of odorized water intake measured in the CCA 120 groups during testing (without US) confirmed this observation. As also shown in Figure 4A, the amount of odorized water intake between conditioning and testing was similar in Sham-lesioned COA 120 group. In contrast, the amount of odorized water decreased between conditioning and testing in EC-lesioned group thus indicating that animals associated the odor with the US with a long ISI. Statistical analyses confirmed these observations and revealed a significant effect of Lesion [*F*_(1, 33)_ = 4.79, *p* < 0.05], a significant interaction between Lesion and Type of procedure [*F*_(1, 33)_ = 6.13, *p* < 0.05], and a significant interaction between Lesion, Type of procedure and Session [*F*_(1, 33)_ = 5.97, *p* < 0.05]. *Post-hoc* comparisons confirmed that odorized water intake was significantly lower during conditioning than during testing in CCA 120 groups (*p* < 0.05) and that the amount of odorized water intake was lower during testing than during conditioning in EC-lesioned COA 120 group (*p* < 0.001). *Post-hoc* comparisons also indicated that, in EC-lesioned COA 120 group, the amount of odorized water intake during testing was lower than in all the other groups (*p* < 0.001 for each comparison).

As shown in Figure 4B, the proportion of time spent in the conditioning context was similar before conditioning and testing in both COA and CCA 120 groups, irrespective of the type of lesion. This suggests that no CCA occurred when the US was administered more than 120 min after the beginning of the context exposure. Figure 4B also showed that the proportion of time spent in the conditioning context seemed higher in CCA than in COA groups, indicative of an initial and maintained higher preference for the conditioning context in CCA 120 groups, as compared to COA 120 groups. Statistical analysis confirmed this observation and revealed a significant effect of Type of procedure [*F*_(1, 33)_ = 10.42, *p* < 0.01], but not of the other factors nor of any interaction.

## Discussion

The results of the present study show that EC lesions induced a deficit in CCA but did not disrupt COA learning; on the contrary, EC-lesioned animals were able to associate the CS with the US even though the ISI was too long to enable sham-lesioned control animals to learn the task. Moreover, the establishment of COA with long ISI obtained in EC-lesioned rats was associated with altered CCA learning.

Sham-lesioned control animals in the CCA group did not display COA with the two intervals tested between LiCl injection and odorized water exposure. In addition, only sham-lesioned control animals that received the US 20 min after the beginning of context exposure (CCA 5) displayed CCA, thus confirming previous findings (Desmedt et al., 2003). These results show that a backward arrangement between CS and US (i.e., no competition between CS and context) leads to CCA but not to COA. Moreover, odorized water intake in the CCA 120 groups was significantly lower than in CCA 5 groups in both sham- and EC-lesioned animals. This result suggests that LiCl affected odorized water intake when injected 60 min before presentation (in CCA 120 groups) but not when it was administered 180 min later (in CCA 5 groups). This temporary interference between LiCl and odorized water intake might reflect a novelty-dependent reaction, the duration of which is limited to the period during which the animal experiences malaise (Domjan, 1977).

Interestingly, the amplitude of the CCA observed in the sham-lesioned COA 5 group was not reduced by simultaneous exposure to the CS. This result suggests that the context was not overshadowed by the CS, and confirms that CCA can occur concomitantly to COA when the CS is presented together with the context during COA learning (Hatfield et al., 1992). On the other hand, the failure to obtain CCA with long ISI might be due to a latent inhibition effect (Lubow and Moore, 1959): previous studies using a conditioned fear paradigm (e.g., Kiernan and Wesbrook, 1993; Killcross et al., 1998) showed that extensive exposure to the to-be-conditioned context resulted in a reduction in contextual fear. Thus, the long exposure (i.e., 140 min) to the conditioning context in the COA 120 and CCA 120 groups may have affected the context-US association by a latent inhibition effect.

Most importantly, the present results show that the EC lesion disrupted CCA. CCA requires learning relations between the different cues present in the learning context, and associating these cues with the US. Since EC-lesioned animals were able to associate the olfactory CS with the US, it is unlikely that the CCA deficit resulted from a failure of US processing. Rather, a large number of studies suggest that it might result from a deficit in context information processing. First, the EC is reciprocally connected to the hippocampus and BLA (e.g., Amaral and Witter, 1995; Ferry et al., 1997; Pitkänen et al., 2000) and it has been previously assumed to be involved in the representation of context (reviews in, e.g., Maren and Fanselow, 1997; Majchrzak et al., 2006; Ji and Maren, 2008; Rudy, 2009; Van Strien et al., 2009; but: Phillips and LeDoux, 1995; Good and Honey, 1997; Bannerman et al., 2001; Hales et al., 2014). The amygdala is a downstream target of the hippocampus for the association of context representation with US (e.g., Fanselow, 2010) and also influences storage of the hippocampus-dependent representation of the conditioning context (Huff and Rudy, 2004; Huff et al., 2005). Moreover, it was recently shown that the glutamatergic projection from BLA to EC (Pitkänen et al., 2000) is involved in the modulation of the acquisition of contextual fear conditioning (Sparta et al., 2014). This suggests that the EC lesion may have impaired CCA through disruption of contextual information processing by both hippocampus and amygdala.

The present results also confirmed that the EC lesion did not disrupt but rather enabled COA, with ISIs up to 120 min (Ferry et al., 1996, 1999, 2006, 2007; Ferry and Di Scala, 1997). Conditioned odor aversion learning (COA) requires association between olfactory CS memory trace and US (Bures and Buresova, 1990; Roldan and Bures, 1994), and we have previously suggested that the EC is involved in the control of olfactory CS memory trace duration through a functional interaction with the BLA (Ferry et al., 1996, 1999; Ferry and Di Scala, 1997). As odor CS and context can both associate with the US in an interdependent way (Rescorla and Wagner, 1972), it is reasonable to suggest that the establishment of COA with long ISI obtained in EC-lesioned animals may have resulted, at least in part, from inhibition of the context influence upon the odor-US association due to the deficit in context processing.

Histological analysis of the lesion extent showed that the aspirative technique damaged a large portion of the lateral EC and part of the medial EC; in the light of previous findings that selective lesion of the lateral but not the medial EC affected COA with long ISI (Ferry et al., 2006), the present effects on COA were likely due to the lesion of the lateral part of the EC. As for the CCA effect, the present results do not indicate which part of the EC was selectively involved. In addition, the aspirative technique induced lesions of axons of passage in the EC and the disruptive effect observed on CCA may have resulted from a deficit in the processing of information arising from or passing through the EC.

Using discrete brain structure inactivation techniques, future studies will probably help to clarify this point, although both parts of the EC seem to be involved in the same kind of mechanism, at least when it comes to spatial processing (Van Cauter et al., 2012).

## Conclusion

Feeding behavior is part of a complex integrated adaptive system. The differentiation between safe and unsafe food items that conditions ingestive behavior depends, at least in part, on previous experience during which the cues characterizing either the food (i.e., odor, taste, texture, etc.) or the environment in which the food is present (contextual cues) acquired a hedonic valence after feeding, through CS-US associative learning. These kinds of association have been experimentally studied for years (Slotnick and Katz, 1974; Nigrosh et al., 1975; Slotnick, 1984) and experimental conditioned food aversion paradigms, such as conditioned taste or odor/taste-potentiated odor aversion learning, have provided fundamental insights into the mechanisms and CNS structures involved in food-reward/food-poisoning associations (see Miranda, 2012 for review). In the case of conditioned aversion learning, numerous studies have shown that context processing influences the strength of the conditioned aversion to a taste acquired in a given context (e.g., Puente et al., 1988; Loy et al., 1993; Skinner et al., 1994; Nakajima et al., 1995; Boakes et al., 1997; Lopez and Cantora, 2003; Murphy and Skinner, 2005; Ishii et al., 2006). Using another type of conditioned food aversion paradigm, the present study clearly shows that the conditions in which COA is established concomitantly to context aversion depends on the time interval separating the presentation of the odor and context from the US. Importantly, the results show that the EC is a key structure in the processes underlying the associations between context, odor CS and US in COA learning. Eventually, our results suggest the EC could be more largely be involved in the acquisition of conditioned food aversion learning through a control upon the association (1) between the odor of a particular food and a gastric malaise (US) that followed its ingestion and (2) between the context in which this food has been encountered and the US.

### Conflict of interest statement

The authors declare that the research was conducted in the absence of any commercial or financial relationships that could be construed as a potential conflict of interest.
